# Genome-Wide Prediction and Validation of Sigma70 Promoters in *Lactobacillus plantarum* WCFS1

**DOI:** 10.1371/journal.pone.0045097

**Published:** 2012-09-20

**Authors:** Tilman J. Todt, Michiel Wels, Roger S. Bongers, Roland S. Siezen, Sacha A. F. T. van Hijum, Michiel Kleerebezem

**Affiliations:** 1 Center for Molecular and Biomolecular Informatics, Nijmegen Center for Molecular Life Sciences, Radboud University Medical Centre, Nijmegen, The Netherlands; 2 HAN University of Applied Sciences, Institute of Applied Sciences, Nijmegen, The Netherlands; 3 NIZO food research, Ede, The Netherlands; 4 TI Food and Nutrition, Wageningen, The Netherlands; 5 Kluyver Centre for Genomics of Industrial Fermentation, Delft, The Netherlands; 6 Wageningen University, Host Microbe Interactomics Group, Wageningen, The Netherlands; 7 Netherlands Bioinformatics Centre, Nijmegen, The Netherlands; Morehouse School of Medicine, United States of America

## Abstract

**Background:**

In prokaryotes, sigma factors are essential for directing the transcription machinery towards promoters. Various sigma factors have been described that recognize, and bind to specific DNA sequence motifs in promoter sequences. The canonical sigma factor σ^70^ is commonly involved in transcription of the cell's housekeeping genes, which is mediated by the conserved σ^70^ promoter sequence motifs. In this study the σ^70^-promoter sequences in *Lactobacillus plantarum* WCFS1 were predicted using a genome-wide analysis. The accuracy of the transcriptionally-active part of this promoter prediction was subsequently evaluated by correlating locations of predicted promoters with transcription start sites inferred from the 5′-ends of transcripts detected by high-resolution tiling array transcriptome datasets.

**Results:**

To identify σ^70^-related promoter sequences, we performed a genome-wide sequence motif scan of the *L. plantarum* WCFS1 genome focussing on the regions upstream of protein-encoding genes. We obtained several highly conserved motifs including those resembling the conserved σ^70^-promoter consensus. Position weight matrices-based models of the recovered σ^70^-promoter sequence motif were employed to identify 3874 motifs with significant similarity (p-value<10^−4^) to the model-motif in the *L. plantarum* genome. Genome-wide transcript information deduced from whole genome tiling-array transcriptome datasets, was used to infer transcription start sites (TSSs) from the 5′-end of transcripts. By this procedure, 1167 putative TSSs were identified that were used to corroborate the transcriptionally active fraction of these predicted promoters. In total, 568 predicted promoters were found in proximity (≤40 nucleotides) of the putative TSSs, showing a highly significant co-occurrence of predicted promoter and TSS (p-value<10^−263^).

**Conclusions:**

High-resolution tiling arrays provide a suitable source to infer TSSs at a genome-wide level, and allow experimental verification of *in silico* predicted promoter sequence motifs.

## Background

Methods for predicting protein-coding genes have been extensively optimized [1], while the development of methodology for the structural and functional analysis of intergenic regions has only come into focus more recently.

Although the fraction of non-coding and/or intergenic sequences in prokaryotic genomes is relatively low compared to eukaryotes [2], it encompasses a similar complexity in conserved DNA sequence motifs and regulatory elements [3]. In prokaryotes, elements such as binding sites for the sigma factor unit of the RNA-polymerase [4] are essential for appropriate guidance of the transcription machinery. Sigma factors enable the binding of the RNA polymerase core enzyme to the canonical promoter sequences they recognize. Various sigma factors have been identified and many bacteria possess more than one sigma factor [5], each recognizing specific DNA sequence motifs in promoters, which enables cells to respond rapidly to changing conditions by sigma factor-mediated adjustment of gene transcription patterns. Although different sigma factors recognize specific promoter sequences, the target sequence elements recognized by particular classes of sigma factors have been reported to be conserved among bacteria [4], [6]. For example, σ^70^ [7], [8] and σ^54^ [9] factors were shown to recognize similar sequences in phylogenetically distant bacterial species. Further fine-tuning of transcription activity by the sigma-factor containing RNA polymerase (e.g., harbouring the canonical σ^70^) involves regulatory factors that either repress or activate transcription by binding to specific DNA sequence motifs located in close proximity of, or overlapping with, the actual promoter sequence [10].

Previously, many studies aimed at detection of intergenic regulatory elements on a genome-wide scale [11], [12] have employed consensus sequences that were deduced from experimentally determined binding sites in order to identify similar regulatory elements elsewhere in the genome [3]. When experimental evidence is limited or absent, comparative genomics or other *de novo* prediction methods can be employed to detect conserved intergenic *cis*-acting elements in a genome [13]–[16].

The species *Lactobacillus plantarum* is frequently encountered in vegetable, meat and dairy fermentations and is also present among the natural inhabitant of mammalian gastrointestinal tracts [17]. The genome of *L. plantarum* WCFS1 [18] was predicted to encode two sigma factors besides σ^70^, namely the *rpoN*-encoded σ^54^ factor that has been shown to exclusively control the expression of the mannose specific PTS system in this strain [9], and the *sigH* encoded sigma factor of which the target genes in *L. plantarum* remain unknown to date, but that resembles the *Bacillus subtilis* 168 transcription factor *sigH* that is involved in sporulation.

In this study, we performed a model-based genome-wide prediction of conserved motifs in the intergenic sequence upstream of protein-encoding genes within the genome of *L. plantarum* WCFS1. *In silico* models of recognizable σ^70^-promoter sequence elements were generated using position weight matrices (PWM; [19], [20]), which were subsequently used for genome-wide prediction of promoter sites. Using this approach we predicted 3874 candidate σ^70^ promoters in the *L. plantarum* WCFS1 genome at p-value cut-offs of 10^−4^. Subsequently, transcriptome datasets obtained using strand-specific genome-wide tiling DNA microarrays were used to validate the transcriptionally active proportion of the *in silico* predicted σ^70^-promoter sequence elements [12], [21], [22], enabling the validation of 568 of the predicted promoters by their proximity (≤40 nucleotides) to 1167 transcription start sites inferred from experimentally estimated transcript boundaries.

## Results

### Prediction of conserved motifs in the intergenic regions of *L. plantarum*

**Figure 1:**
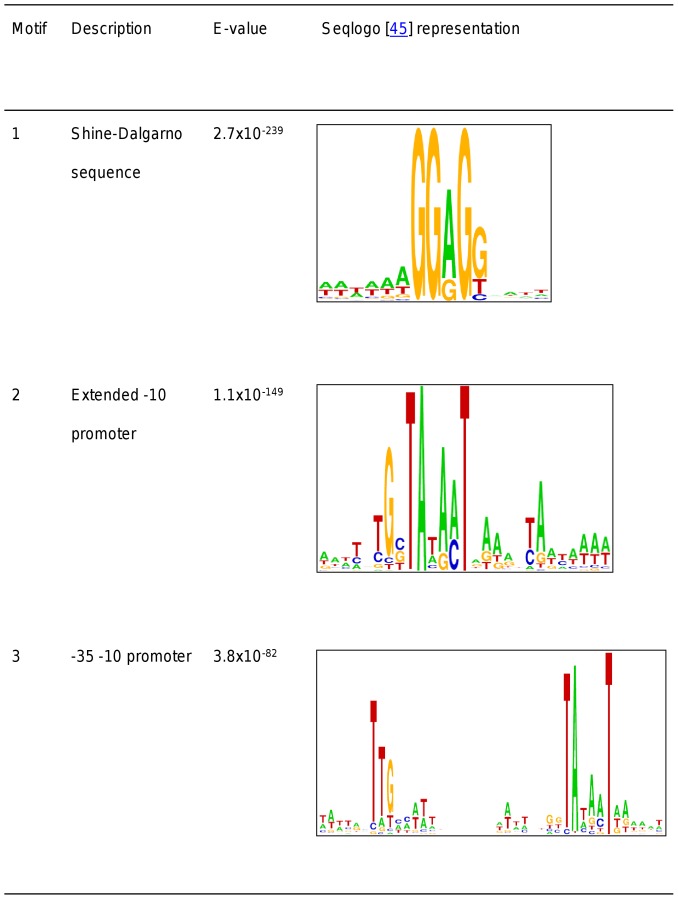
Highly conserved motifs. Highly conserved motifs (E-value<10^−80^) detected by MEME search in the upstream regions of protein-encoding genes (for a full list see Figure S1 in Supplementary results). Motifs are shown as Seqlogos [45].

With 3.3 million nucleotides, the genome of *L. plantarum* WCFS1 is among the largest *Lactobacillus* genomes [18] and comprises 3137 predicted genes, of which 3051 are protein-encoding (Refseq NC_004567, [23]). The intergenic regions encompass approximately 16 percent of the entire genome nucleotide sequence (530640 out of 3308275 nucleotides). Using the genome annotation of *L. plantarum* WCFS1, 2209 intergenic sequences (see Methods) were used to search for conserved motifs using MEME [24], resulting in the detection of eight distinct sequence motifs with E-values below 10^−3^ (see Figure 1, Results S1 and Figure S1).

The element with the lowest E-value (motif 1; E-value 2.7×10^−239^; Figure 1) displayed clear resemblance to the consensus Shine and Dalgarno sequence (SD; 5′-AGAAAGGAGGTGATC-3′) of bacteria [25]. The SD sequence of *L. plantarum* WCFS1 is found because the intergenic sequences used to search for conserved motifs generally include the untranslated regions of the protein-encoding genes that encompass the SD sequence. In addition to the SD sequence, two motifs (motif 2, motif 3; Figure 1) were identified that resemble *cis*-acting elements described for sigma factor σ^70^ (*rpoD*) [6] dependent promoters. These motifs encompassed one or both of the two canonical consensus σ^70^ elements that were previously designated the −35 and −10 box (consensus sequence 5′-TTGACA-3′and 5′-TATAAT-3′, respectively) [4]. Both motif 2 (E-value 1.1×10^−149^) and motif 3 (E-value 3.8×10^−82^) encompass sequences that resemble the −10 box, while motif 3 also includes the −35 box.

### Genome-wide prediction of σ^70^ promoters in *L. plantarum*

The detection of *L. plantarum* related σ^70^-promoter elements enabled the genome-wide prediction of σ^70^-promoters that share similarity with the identified motifs. The two σ^70^-promoter associated position-specific weight matrices (PWMs) generated by MEME (motif 2 and motifs 3) were employed to predict the positions of both sequence elements in the *L. plantarum* WCFS1 chromosome, using MAST [26]. These predictions generated 939 and 1141 occurrences of the extended −10 (based on motif 2) and the −35 −10 promoter motifs (based on motif 3) at a p-value cut-off of 10^−4^, respectively. Occurrences were filtered for partially overlapping promoters, resulting in the identification of 1879 putative σ^70^ promoters. Previous studies have revealed that the two σ^70^-promoter elements (−35 and −10 boxes) may flank a spacer sequence of slightly variable length in different promoters (16–20 nt) [27]. To accommodate such spacer-length variation in our predictions, the PWM of the −35 −10 promoter (based on motif 3) was manually modified to allow for length variations ranging from 16 to 22 nt. MAST-based searches using these modified PWMs yielded 1995 additional non-overlapping predicted promoter sequences. Overall, these predictions generated a total of 3874 putative promoter sequences that displayed high-level similarity to the consensus motif (Bonferroni corrected p-values<0.05).

### Detection of putative transcription start sites

As described in previous studies, the high sensitivity of *in silico* prediction of σ^70^-promoters using PWMs usually generates many false-positive identifications [3], [28]–[30]. To overcome this limitation, putative transcription start sites (TSSs) inferred from 5′-ends of transcripts are commonly employed to corroborate predicted σ^70^-promoter locations [12], [21], [31]. In this study, we determined genome-wide putative TSSs from tiling-array derived transcription data obtained for *L. plantarum* WCFS1 grown in rich laboratory medium (MRS) at 37°C. Transcriptional activity was assigned to genomic regions by employing a global threshold approach [32], leading to the identification of 1167 distinct transcriptionally active regions (TARs) larger than 120 nucleotides [33]. The 1167 TARs cover 1995 of the 3137 predicted genes in the *L. plantarum* WCFS1 genome, likely reflecting the transcriptionally active fraction of the entire genomic coding capacity under the growth conditions employed in this study.

The 5′-ends of TARs may be the result of transcription initiation but can also arise from other events, such as degradation, processing or cross-hybridisation. Although in the majority of cases 5′-end points of TARs are probably the TSSs [12] false-positive identifications are possible (see Results S2, Figure S2 and S3). Inspection of signal strength patterns at the 5′-end of different TARs, revealed sharper increases in signal intensity for highly expressed TARs compared to weakly expressed TARs. Consequently, the assignment of putative TSSs to weakly expressed TARs leads to more ambiguous TSS positioning as compared to those assigned to highly expressed TARs. Therefore, TARs were grouped according to their mean signal intensity (see Methods), which allowed the discrimination between TSSs assigned to highly and weakly expressed TARs. To consistently assign TSSs to TARs an algorithm was developed that identifies the maximum increase in signal intensity at the 5′-end of a TAR and by linear extrapolation of the local transcription signal-intensity slope estimates the position of the TSS of the TAR. We evaluated the performance of our TSS predictions by correlating the tiling array-based TSSs with those that were previously identified in *L. plantarum* by primer extension. For nearly all cases (11 out of 12 genes) the putative TSS predicted on basis of the tiling array data was within a maximum distance of 28 nt from the experimentally TSS determined by primer extension (see Results S3). The majority of these genes (8 genes) were highly expressed in the tiling array dataset (their average expression signal belonged to the two groups of TARs that showed the highest expression levels, see Method).

**Figure 2:**
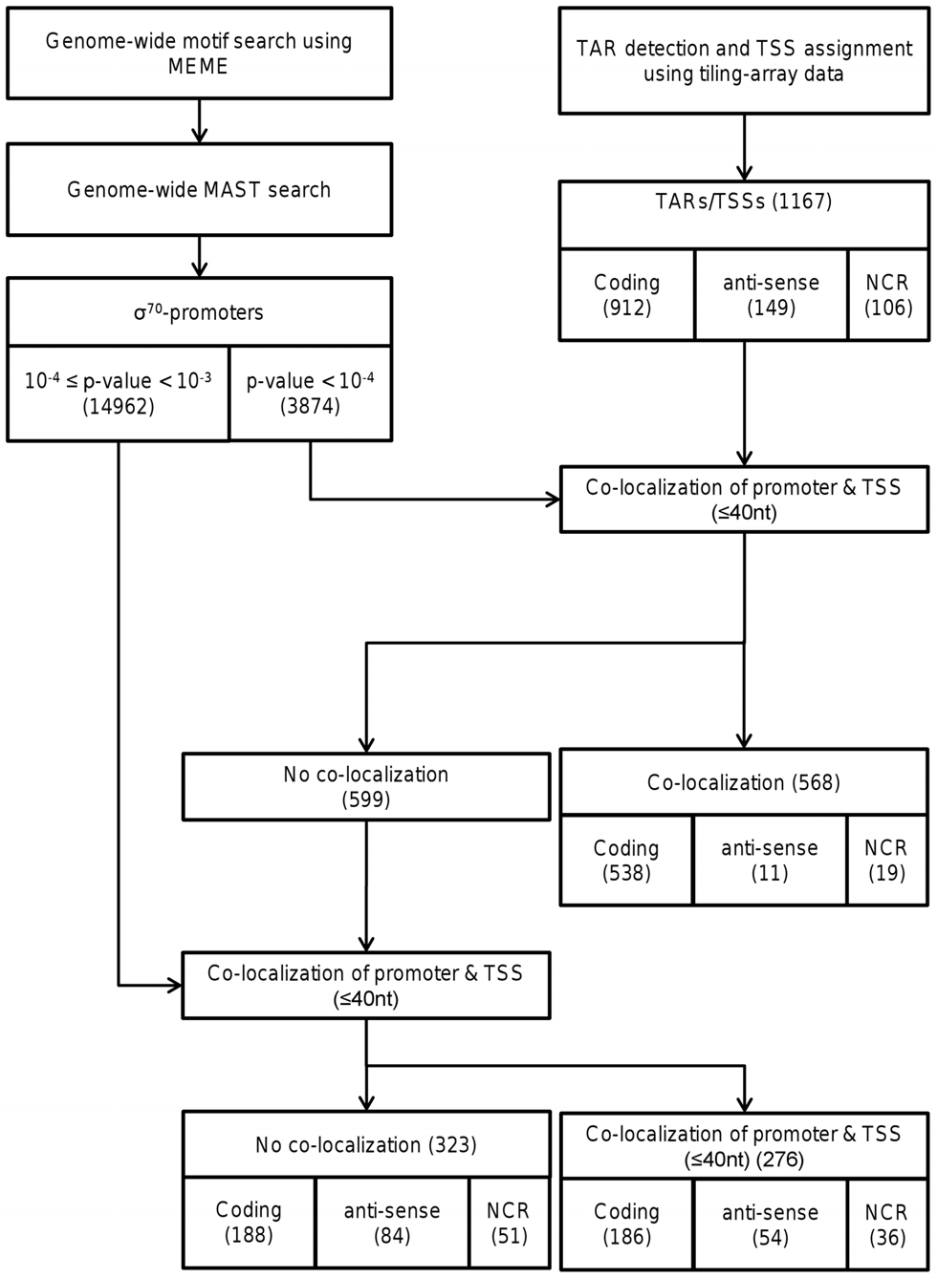
Flow chart of promoter validation. Genome-wide motif search using MEME and MAST provided 3874 and 14962 predicted σ^70^-promoters at p-value cut-offs of 10^−4^ and (10^−4^≤p-value<10^−3^), respectively. For validation of the predicted promoters, transcriptionally active regions (TARs) were detected using tiling array data of *L. plantarum* WCFS1 and for each TAR the transcription start site (TSS) was determined. After filtering the TSSs for low-expressed TARs, 621 TSSs were obtained for validation of predicted σ^70^-promoters. At a low p-value cut-off (p-value<10^−4^), 568 σ^70^-promoters were found that co-localized within a distance of 40 nt of TSSs. An additional 120 σ^70^-promoters were found to co-localized within a distance of 40 nt of TSSs after raising the p-value cut-off (10^−4^≤p-value<10^−3^). For 94 TSSs, no co-localized σ^70^-promoter (within a distance of 40 nt) was found.

Application of the algorithm allowed prediction of a TSS for each of the 1167 TARs. Expectedly, out of the 1167 predicted TSSs, the majority were located upstream of protein encoding genes (912). Next to these expected TSSs, 149 of the predicted TSSs appeared to be positioned in an antisense orientation relative to the annotated genes, while an additional 106 predicted TSSs were positioned in genomic regions that lack genetically linked genes, suggesting that these TSSs could represent the start of non-coding RNAs (see Figure 2).

### Validation of predicted σ^70^ promoters using TSSs

**Figure 3:**
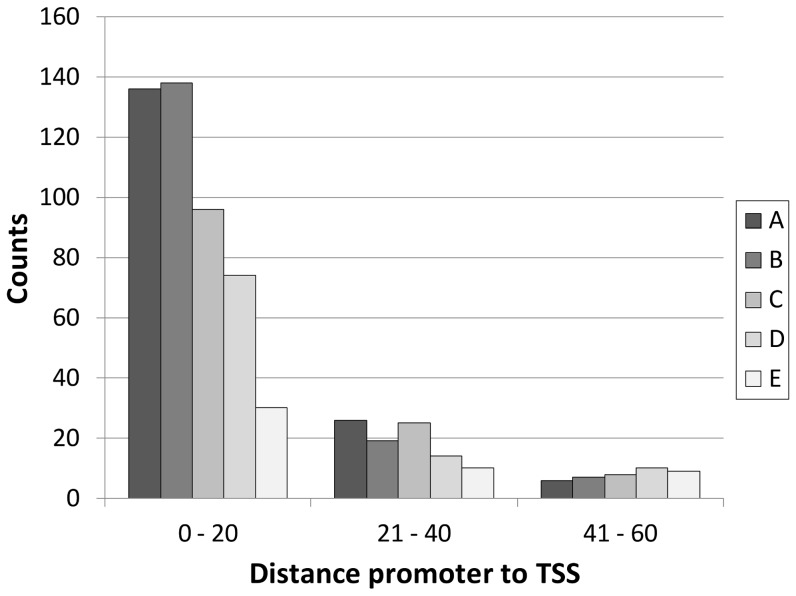
Distribution of distances between promoters and TSSs. Distribution of distances (in nt) for 568 cases where a predicted σ^70^-promoter (genome-wide search, p-values<10^−4^) and a TSS were found in proximity (≤60 nt) of each other. TSSs are divided into 5 groups (A, B, C, D, E) defined by the differences between the means of signal intensities upstream and downstream of the TSS. The 5 groups are the inter-percentile ranges (E: 0–20%, D: 21–40%, C:41–60%, B: 61–80%, A: 81–100%) of the ranked mean differences (see Methods). At distances above 40 nt the observed number of co-localized TSSs and promoters (≤10 counts) is equal to the expected number co-localized TSSs and promoters (see Methods).

**Figure 4:**
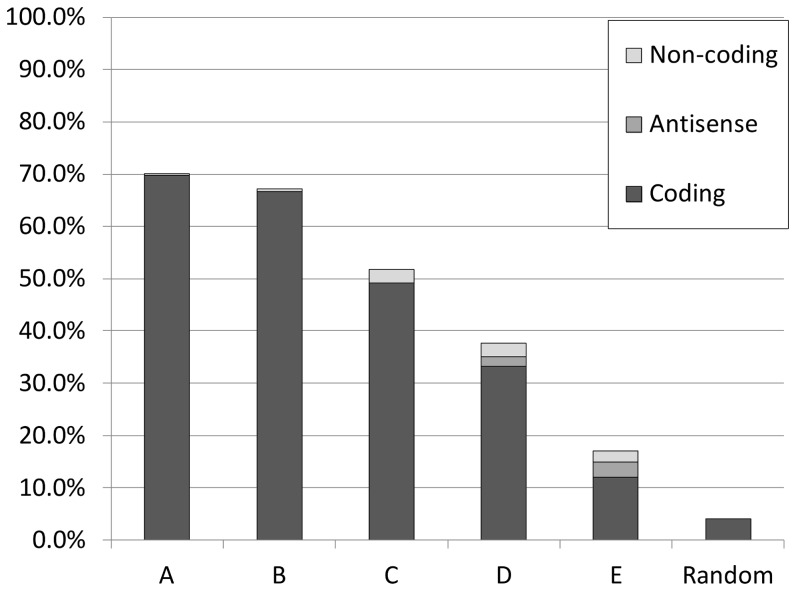
Frequency distribution of validated promoters. Frequency distribution of σ^70^-promoters (genome-wide search, p-values<10^−4^) with TSSs found in proximity (≤40 nt) of each other. Frequencies are given for 5 groups (A, B, C, D, E) and the expected frequency of co-localized TSSs and promoters (Random). The 5 groups are the inter-percentile ranges (E: 0–20%, D: 21–40%, C:41–60%, B: 61–80%, A: 81–100%) of the ranked mean differences between the means of signal intensities upstream and downstream of the TSS (see Methods).

Experimentally verified TSSs are commonly located between 8–14 nucleotides downstream of their cognate promoter −10 sequence element [3]. Therefore, the positions of inferred TSSs and their relative distance to predicted promoters can be used to corroborate the involvement of these promoters in the initiation of transcription of the corresponding TAR. The TSS locations were correlated to appropriately oriented predicted σ^70^-promoters (p-value<10^−4^) in close proximity of the TSS, identifying 568 σ^70^-promoters within a distance 40 nt of a TSS (see Figure 2) of which 474 σ^70^-promoters were within a distance 20 nt of a TSS (tiling-array resolution 14 nt) (see Figure 3). About 70% of TSSs assigned to highly expressed TARs (group A and B in Figure 4) were in close proximity of a predicted σ^70^-promoter (≤40 nt), while for ‘only’ 17% of TSSs detected for low expressed TARs (group E in Figure 4) a σ^70^-promoter could be assigned. Expectedly, TSSs inferred from expression signals of highly expressed genes give better corroboration results than TSSs inferred from expression signals of lower expressed genes. The lower promoter assignment to the low-expression TARs may be due to the ambiguity of the TSS prediction for these TARs, while the higher accuracy of TSS prediction for high expression TARs provides more confident co-localizations with corresponding promoter predictions. Overall, TSSs and predicted promoters displayed significant co-occurrence (Fisher exact p-value<10^−263^) based on the detected TSSs matching with predicted promoters in comparison with randomly generated TSSs. The expected co-occurrence frequencies of TSSs and promoters within a range of 40 nucleotides are predicted to be 4% (see Figure 4, group Random).

As σ^70^ target sequences may exhibit quite extensive sequence variation, this approach may have missed recognizable, but less conserved, σ^70^-promoter sequence elements. To expand our analyses to also encompass σ^70^-promoters with more deviating sequences, the stringency of promoter detection was relieved (10^−4^≤p-value<10^−3^), allowing MAST-based detection of an additional 14962 sequence elements with σ^70^-promoters similarity. Of these predicted elements 276 co-localized within a distance of 40 nt of TSSs for which no conserved σ^70^ promoters (p-value<10^−4^) could be found (see Figure 2 and Figure S4). However, TSSs and these lower stringency predicted promoters displayed no significant co-occurrence (Fisher exact p-value = 0.16) and co-occurrence frequencies of TSSs and promoters within a range of 40 nucleotides are predicted to be 19% (see Figure S5).

## Discussion

In this study we determined the consensus motifs of the σ^70^ promoters in *L. plantarum* WCFS1 using different models and approaches, including the validation of predicted promoters using tiling array transcriptome datasets.

Searching for conserved motifs in the intergenic regions of *L. plantarum* WCFS1 revealed highly conserved motifs (see Figure 1). Analogous to their anticipated sequence conservation, the two elements of the general σ^70^ promoter, the so-called −35 and −10 elements, were among the most conserved motifs (p-values<10^−4^).

The σ^70^ promoter related motifs were used to generate position weight matrix (PWM) based models. PWM models enabled the genome-wide detection of 3874 and additional 14962 putative σ^70^ promoters in the *L. plantarum* WCFS1 genome at p-value cut-offs of 10^−4^ and 10^−3^, respectively. Removing all σ^70^ promoters that were intergenic but overlapped partly with the 5′- or 3′-ends of genes yielded 1763 and 4093 σ^70^ promoters at p-value cut-offs of 10^−4^ and 10^−3^, respectively. As shown in previous studies on lactic acid bacteria [16], the average number of transcriptional units is about 1077 with an average of 1.77 genes per unit suggesting that the number of σ^70^ promoters reflects the number of transcripts that would be expected on basis of the number of predicted operons on the genome of *L. plantarum* [16]. The additional σ^70^ promoters partly overlapping with coding parts of genes might be alternative σ^70^ promoters (see for example Results S2) which are also found in other bacterial genomes [34].

PWMs have been used to predict σ^70^-promoters with high sensitivity in other species [3], [35], [36], but were hampered by many false-positive identifications [3], [28]–[30], illustrating the difficulty to model the general σ^70^-promoter sequence elements, which are bipartite motifs composed of 2 conserved sequence motifs separated by a non-conserved spacer sequences of variable length [27], [37], [38].

Analogous to what was described in previous studies [12], [21], [31], transcription start sites (TSS), determined by high-resolution tiling arrays, can be used to corroborate predicted σ^70^-promoter locations. The analysis of transcriptome datasets from *L. plantarum* WCFS1 obtained with strand-specific genome-wide tiling arrays revealed 1167 transcriptionally active regions (TARs). The 5′-ends of TARs were used to infer putative TSSs (see Figure 2). To confirm the TSSs inferred from the 5′-ends of TARs these TSSs were compared to TSSs that have previously been determined by primer extension analysis (see Results S3). Our predicted TSSs co-localised nicely with the previously described TSS positions. With the available TSS information, we were able to show that our approach gives highly reliable results. Similarly, alternative genome wide transcription analysis methods such as RNA-seq can also be employed to detect TSSs in bacteria [31], [39], [40], but here we confirm that high-resolution tiling array data are a valuable resource to infer TSS locations [12], [21], [22].

Of the 1167 putative TSSs selected for validation, 568 TSS were positioned within proximity of the σ^70^ promoters that were predicted with high stringency, while an additional 276 TSSs could be co-localized with predicted promoters using the lower stringency promoter prediction (10^−4^≤p-values<10^−3^). However, the latter co-occurrence of TSSs and low-stringency predicted promoters was not significant (Fisher exact p-value = 0.16), which is likely due to a substantial overprediction (false-positives) of promoter sequences.

In general, promoters function more efficiently with sequence elements that resemble the consensus sequences. However, most promoters have non-consensus sequences and their activity is further modulated by trans-acting factors (including alternative sigma factors, small ligands and transcription factors) [34]. To discover conserved transcription initiation motifs other than the σ^70^ promoter-related sequences, DNA sequences upstream to TSSs with no σ^70^ promoter were searched for other conserved motifs (applying MEME searches as described for the initial search, see Method). These searches did not reveal additional highly-conserved motifs (p-value cut-off<10^−4^; data not shown).

Our results show that motif-based approaches can be used to recover conserved sequence elements of the σ^70^ promoter. However, the discovery of more degenerate promoter elements is hampered by many false-positive identifications [3], [28]–[30] and validation of predicted promoters (e.g. using transcriptome data) is required but limited by the statistical significance of the obtained results. Our findings suggest that these more degenerate σ^70^ promoters may constitute a considerable proportion of all σ^70^ promoters in a given genome (in our case about 24% of the validated σ^70^ promoters have p-values≥10^−4^), which has been described for other bacterial genomes (such as *E. coli*) [34]. In this study, transcription data under a single growth condition was used limiting the validation to the active portion of σ^70^ promoter-related genes that are clearly transcribed. Transcription data obtained under a variety of growth conditions would still be required to validate the full complement of functional σ70 promoters and distinguish them from false-positive predictions.

## Materials and Methods

### Transcription analysis of *Lactobacillus plantarum* WCFS1

For genome wide transcriptome analysis *L. plantarum* WCFS1 [18] was grown until mid-exponential growth phase in MRS, cells were harvested and RNA was isolated and labeled as described before [41], [42]. 1.5 µg of labeled targets were hybridized and analyzed on WCFS1 DNA microarrays that cover the whole genome in a tiling fashion as described (GSE36898, GPL15385, GPL15386). The gene expression data are MIAME compliant and deposited in the gene expression omnibus (GEO) database (accession numbers GSE36898, GPL15385, GPL15386).

The hybridization intensities of two biological replicates were obtained. Hybridization intensities were log2-transformed, background corrected and normalized by quantile normalization [43]. After normalization, intensities of the two replicates were averaged yielding two strand-specific transcript-level data sets.

### Building σ^70^-promoter models using PWMs

Position weight matrices were constructed from high-scoring motifs identified by MEME [24] searches on a selected subset of intergenic DNA sequences of the *L. plantarum* WCFS1 genome. Initially, all 100 nt regions upstream of all protein encoding genes were selected from the *L. plantarum* WCFS1 genome (Refseq NC_004567). Subsequently, these sequences were evaluated for their potential overlap with a preceding gene, and in such cases, only the intergenic sequence was used for analysis, provided they were 25 nt or longer.

The resulting set of selected intergenic sequences was searched for conserved motifs using MEME [24], applying standard DNA parameter settings. Only motifs reported by MEME with E-values below 10^−4^ were considered relevant for further analysis. Additional parameters used for selection of candidate sequences were: 1) Zero or One Occurrence Per Sequence (ZOOPS mode), 2) a maximum of ten different motifs per sequence, and 3) each motif should be found in at least thirty-five different sequences. PWM models were constructed for the most abundantly encountered motifs, including those resembling the canonical −35 and −10 elements known from general σ^70^-dependent promoters [6].

### Predicting σ^70^ promoters

The PWM models were used in a MAST [26] search against the complete genomic sequence of *L. plantarum* WCFS1 including the coding regions to predict potential σ^70^ promoters. MAST hits with relatively high significance (p-values<10^−4^) were used in further analysis, unless indicated otherwise.

Because the spacer region between the −35 and −10 boxes of the σ^70^-promoter varies in length (16–20 nt) [27], PWM models were manually altered by either copying or deleting the columns in the position weight matrix that were part of the spacer region and had the least functional information (low overall specificity and low sequence conservation) of this region. These new matrices were used in genome-wide MAST searches to predict additional promoter occurrences with different spacer-lengths. All predicted promoters (including those predicted on basis of the extended −10 box motif) were filtered for overlapping promoter elements. In case of overlap, the hit with the highest significance was used for further analysis. All non-intergenic promoter elements were removed.

### Detecting transcriptionally active regions

A global threshold approach was used to detect significantly transcribed genomic regions. For each strand-specific dataset the global threshold was determined from the log distribution of the probe signals. A background normal distribution was estimated from the left-of-the-mode data [43] and used to estimate the background probability of a probe signal. The global threshold was set to a signal level at which the probability of a probe signal being a background signal was less than 10^−6^.

Next, transcriptionally active regions (TARs; [33]) were determined by applying a segmentation algorithm (comparable to transfrags as described in [32] and [44]) using the previously estimated global threshold. Segments of adjacent probes with signal intensities above the threshold were identified. Adjacent segments with a gap of maximal 60 nt (corresponding to the length of a single probe on the tiled micro-array) were joined and, segments shorter than 120 nt were discarded.

### Transcription start site determination and statistical analysis

The 5′-end of a TAR was used to estimate the corresponding TSS position. Manual inspection of signal patterns of different TARs in the tiling array data revealed that highly expressed regions display a rather sharp, approximately linear rise in signal intensity while in lowly expressed regions the signal change appeared to be sigmoidal (data not shown). Based on this observation, we developed the following procedure to determine TSS: Probe signals from the 5′-end of a TAR and its upstream region were used to determine the genomic position at which the slope of increase in signal intensities reached its maximum. This position was used to linearly extrapolate the position of the TSS using the difference between the probe signal of the maximum slope position and the background signal (derived from the mode of the distribution of the probe signals).

Using the gene annotation of *L. plantarum* WCFS1 (Refseq NC_004567, [23]), TSSs were labelled anti sense if the transcribed strand (TAR) was opposite to a gene-containing strand. In addition, if a TAR had no overlap with known genes (based on the current gene annotation) the assigned TSS and its corresponding TAR were annotated as non-coding. All other TSSs were assigned to the closest downstream located gene positioned on the same strand.

To estimate the probability for randomly selecting a TSS in a 40 nucleotide range from a promoter, we used the genomic positions of the TSSs, randomly selected genomics positions of promoters (10.000 times) and calculated for each TSS the minimum distance to the 3′end of a promoter.

TSSs were grouped according to the increase in signal intensity by (1) calculating the differences between the means of expression signal upstream and downstream of the TSS and (2) division of the TSS into five groups according to their rank position (inter-percentile ranges 0–20%, 21–40%, 41–60%, 61–80% and 81–100%).

## Supporting Information

Results S1 Conserved motifs detected by MEME in the upstream regions of protein-encoding genes in *L. plantarum* WCFS1.
(DOCX)

Results S2 Interpretation of TAR detection results.
(DOCX)

Results S3 Evaluation of results of the TSS prediction method.
(DOCX)

Figure S1 **Motifs detected by MEME in** ***L. plantarum*** **WCFS1.** Motifs are displayed as Seqlogo's that contain at each position stacks of letters. The height of an individual letter in a stack represents the probability of the letter at that position in an occurrence of the motif. The position-specific probabilities are retrieved from the position-specific weight matrices calculated by MEME.
(TIF)

Figure S2 **Inferred signal intensity of a genomic region containing two genes.** Inferred signal intensity plotted on part of the *L. plantarum* WCFS1 chromosome (genome location: 1156200–1159500 of the forward strand). At this location two genes are located (*lp_1273* and *lp_1274*) which are considered to form an operon. The tiling microarray signal intensities measured confirms that both genes are transcribed at an approximately equal level and suggest that the inferred transcription start is 537 nucleotides upstream of *lp_1273*. The black box indicates the position of a predicted σ^70^-promoter. The grey box indicates the position of a second predicted σ^70^-promoter with no close TSS.
(TIF)

Figure S3 **Inferred signal intensity of a genomic region containing two genes.** Inferred signal intensity plotted on part of the *L. plantarum* WCFS1 chromosome (genome location: 1407149–1410217 of the forward strand). At this location two genes are located (*lp_1541* and *lp_1543*) expressed at different levels. For *lp_1541* a putative TSS and a predicted σ^70^-promoter co-localized (2 nt apart) upstream to the annotated translation start of the gene. For *lp_1543* a putative TSS is located 145 nt downstream of the annotated translation start of *lp_1543* and a σ^70^-promoter is predicted 31 nt upstream of this translation start. The low signal expression of *lp_1543* makes it difficult to infer a clear TSS position. The black boxes indicate the positions of predicted σ^70^-promoters.
(TIF)

Figure S4 **Distribution of distances between promoters and TSSs.** Distribution of distances (in nt) for 568 cases where a predicted σ^70^-promoter (10^−4^≤p-values<10^−3^) and a TSS were found in proximity (≤60 nt) of each other. TSSs are divided into 5 groups (A, B, C, D, E) defined by the differences between the means of signal intensities upstream and downstream of the TSS. The 5 groups are the inter-percentile ranges (E: 0–20%, D: 21–40%, C:41–60%, B: 61–80%, A: 81–100%) of the ranked mean differences (see Methods). At distances above 40 nt the observed number of co-localized TSSs and promoters (≤10 counts) is equal to the expected number co-localized TSSs and promoters (see Methods).
(TIF)

Figure S5 **Frequency distribution of validated promoters.** Frequency distribution of σ^70^-promoters (10^−4^≤p-values<10^−3^) with TSSs found in proximity (≤40 nt) of each other. Frequencies are given for 5 groups (A, B, C, D, E) and the expected frequency of co-localized TSSs and promoters (Random). The 5 groups are the inter-percentile ranges (E: 0–20%, D: 21–40%, C:41–60%, B: 61–80%, A: 81–100%) of the ranked mean differences between the means of signal intensities upstream and downstream of the TSS (see Methods).
(TIF)

## References

[1] Baxevanis AD (2004) An overview of gene identification: approaches, strategies, and considerations. Curr Protoc Bioinformatics Chapter 4: Unit4 1. 10.1002/0471250953.bi0401s6 18428724

[2] Mira A, Ochman H, Moran NA (2001) Deletional bias and the evolution of bacterial genomes. Trends Genet 17: 589–596. 11585665 10.1016/s0168-9525(01)02447-7

[3] Huerta AM, Collado-Vides J (2003) Sigma70 promoters in *Escherichia coli*: specific transcription in dense regions of overlapping promoter-like signals. J Mol Biol 333: 261–278. 14529615 10.1016/j.jmb.2003.07.017

[4] Gruber TM, Gross CA (2003) Multiple sigma subunits and the partitioning of bacterial transcription space. Annu Rev Microbiol 57: 441–466. 14527287 10.1146/annurev.micro.57.030502.090913

[5] Kroos L, Yu YT (2000) Regulation of sigma factor activity during *Bacillus subtilis* development. Current opinion in microbiology 3: 553–560. 11121773 10.1016/s1369-5274(00)00140-5

[6] deHaseth PL, Zupancic ML, Record MT Jr (1998) RNA polymerase-promoter interactions: the comings and goings of RNA polymerase. J Bacteriol 180: 3019–3025. 9620948 10.1128/jb.180.12.3019-3025.1998 PMC107799

[7] Lonetto M, Gribskov M, Gross CA (1992) The sigma 70 family: sequence conservation and evolutionary relationships. J Bacteriol 174: 3843–3849. 1597408 10.1128/jb.174.12.3843-3849.1992 PMC206090

[8] Paget MS, Helmann JD (2003) The sigma70 family of sigma factors. Genome Biol 4: 203. 12540296 10.1186/gb-2003-4-1-203 PMC151288

[9] Stevens MJ, Molenaar D, de Jong A, De Vos WM, Kleerebezem M (2010) sigma54-Mediated control of the mannose phosphotransferase sytem in *Lactobacillus plantarum* impacts on carbohydrate metabolism. Microbiology 156: 695–707. 19942662 10.1099/mic.0.034165-0

[10] Lloyd G, Landini P, Busby S (2001) Activation and repression of transcription initiation in bacteria. Essays Biochem 37: 17–31. 11758454 10.1042/bse0370017

[11] Down TA, Bergman CM, Su J, Hubbard TJ (2007) Large-scale discovery of promoter motifs in *Drosophila melanogaster* . PLoS Comput Biol 3: e7. 17238282 10.1371/journal.pcbi.0030007 PMC1779301

[12] McGrath PT, Lee H, Zhang L, Iniesta AA, Hottes AK, et al (2007) High-throughput identification of transcription start sites, conserved promoter motifs and predicted regulons. Nat Biotechnol 25: 584–592. 17401361 10.1038/nbt1294

[13] Alkema WB, Lenhard B, Wasserman WW (2004) Regulog analysis: detection of conserved regulatory networks across bacteria: application to *Staphylococcus aureus* . Genome Res 14: 1362–1373. 15231752 10.1101/gr.2242604 PMC442153

[14] McGuire AM, Hughes JD, Church GM (2000) Conservation of DNA regulatory motifs and discovery of new motifs in microbial genomes. Genome Res 10: 744–757. 10854408 10.1101/gr.10.6.744

[15] van Nimwegen E, Zavolan M, Rajewsky N, Siggia ED (2002) Probabilistic clustering of sequences: inferring new bacterial regulons by comparative genomics. Proc Natl Acad Sci U S A 99: 7323–7328. 12032281 10.1073/pnas.112690399 PMC124229

[16] Wels M, Francke C, Kerkhoven R, Kleerebezem M, Siezen RJ (2006) Predicting cis-acting elements of *Lactobacillus plantarum* by comparative genomics with different taxonomic subgroups. Nucleic Acids Res 34: 1947–1958. 16614445 10.1093/nar/gkl138 PMC1435977

[17] Ahrne S, Nobaek S, Jeppsson B, Adlerberth I, Wold AE, et al (1998) The normal *Lactobacillus* flora of healthy human rectal and oral mucosa. Journal of applied microbiology 85: 88–94. 9721659 10.1046/j.1365-2672.1998.00480.x

[18] Kleerebezem M, Boekhorst J, van Kranenburg R, Molenaar D, Kuipers OP, et al (2003) Complete genome sequence of *Lactobacillus plantarum* WCFS1. Proc Natl Acad Sci U S A 100: 1990–1995. 12566566 10.1073/pnas.0337704100 PMC149946

[19] Stormo GD (2000) Gene-finding approaches for eukaryotes. Genome research 10: 394–397. 10779479 10.1101/gr.10.4.394

[20] Staron A, Sofia HJ, Dietrich S, Ulrich LE, Liesegang H, et al (2009) The third pillar of bacterial signal transduction: classification of the extracytoplasmic function (ECF) sigma factor protein family. Molecular microbiology 74: 557–581. 19737356 10.1111/j.1365-2958.2009.06870.x

[21] Tjaden B, Saxena RM, Stolyar S, Haynor DR, Kolker E, et al (2002) Transcriptome analysis of *Escherichia coli* using high-density oligonucleotide probe arrays. Nucleic Acids Res 30: 3732–3738. 12202758 10.1093/nar/gkf505 PMC137427

[22] Selinger DW, Cheung KJ, Mei R, Johansson EM, Richmond CS, et al (2000) RNA expression analysis using a 30 base pair resolution *Escherichia coli* genome array. Nat Biotechnol 18: 1262–1268. 11101804 10.1038/82367

[23] Siezen RJ, Francke C, Renckens B, Boekhorst J, Wels M, et al (2012) Complete resequencing and reannotation of the *Lactobacillus plantarum* WCFS1 genome. J Bacteriol 194: 195–196. 22156394 10.1128/JB.06275-11 PMC3256602

[24] Bailey TL, Elkan C (1994) Fitting a mixture model by expectation maximization to discover motifs in biopolymers. Proc Int Conf Intell Syst Mol Biol 2: 28–36. 7584402

[25] Shine J, Dalgarno L (1975) Determinant of cistron specificity in bacterial ribosomes. Nature 254: 34–38. 803646 10.1038/254034a0

[26] Bailey TL, Gribskov M (1998) Methods and statistics for combining motif match scores. Journal of computational biology : a journal of computational molecular cell biology 5: 211–221. 9672829 10.1089/cmb.1998.5.211

[27] Helmann JD (1995) Compilation and analysis of *Bacillus subtilis* sigma A-dependent promoter sequences: evidence for extended contact between RNA polymerase and upstream promoter DNA. Nucleic Acids Res 23: 2351–2360. 7630711 10.1093/nar/23.13.2351 PMC307037

[28] Fickett JW, Hatzigeorgiou AG (1997) Eukaryotic promoter recognition. Genome Res 7: 861–878. 9314492 10.1101/gr.7.9.861

[29] Kanhere A, Bansal M (2005) A novel method for prokaryotic promoter prediction based on DNA stability. BMC Bioinformatics 6: 1. 15631638 10.1186/1471-2105-6-1 PMC545949

[30] Pedersen AG, Baldi P, Chauvin Y, Brunak S (1999) The biology of eukaryotic promoter prediction–a review. Comput Chem 23: 191–207. 10404615 10.1016/s0097-8485(99)00015-7

[31] Mendoza-Vargas A, Olvera L, Olvera M, Grande R, Vega-Alvarado L, et al (2009) Genome-wide identification of transcription start sites, promoters and transcription factor binding sites in *E. coli* . PLoS One 4: e7526. 19838305 10.1371/journal.pone.0007526 PMC2760140

[32] Cheng J, Kapranov P, Drenkow J, Dike S, Brubaker S, et al (2005) Transcriptional maps of 10 human chromosomes at 5-nucleotide resolution. Science 308: 1149–1154. 15790807 10.1126/science.1108625

[33] Rinn JL, Euskirchen G, Bertone P, Martone R, Luscombe NM, et al (2003) The transcriptional activity of human Chromosome 22. Genes & development 17: 529–540. 12600945 10.1101/gad.1055203 PMC195998

[34] Browning DF, Busby SJ (2004) The regulation of bacterial transcription initiation. Nat Rev Microbiol 2: 57–65. 15035009 10.1038/nrmicro787

[35] Hertz GZ, Stormo GD (1996) *Escherichia coli* promoter sequences: analysis and prediction. Methods in enzymology 273: 30–42. 8791597 10.1016/s0076-6879(96)73004-5

[36] Mulligan ME, Hawley DK, Entriken R, McClure WR (1984) *Escherichia coli* promoter sequences predict in vitro RNA polymerase selectivity. Nucleic Acids Res 12: 789–800. 6364042 10.1093/nar/12.1part2.789 PMC321093

[37] Lisser S, Margalit H (1993) Compilation of E. coli mRNA promoter sequences. Nucleic Acids Res 21: 1507–1516. 8479900 10.1093/nar/21.7.1507 PMC309355

[38] Ozoline ON, Deev AA, Arkhipova MV (1997) Non-canonical sequence elements in the promoter structure. Cluster analysis of promoters recognized by *Escherichia coli* RNA polymerase. Nucleic Acids Res 25: 4703–4709. 9365247 10.1093/nar/25.23.4703 PMC147123

[39] Sharma CM, Hoffmann S, Darfeuille F, Reignier J, Findeiss S, et al (2010) The primary transcriptome of the major human pathogen *Helicobacter pylori* . Nature 464: 250–255. 20164839 10.1038/nature08756

[40] Guell M, van Noort V, Yus E, Chen WH, Leigh-Bell J, et al (2009) Transcriptome complexity in a genome-reduced bacterium. Science 326: 1268–1271. 19965477 10.1126/science.1176951

[41] Marco ML, Peters TH, Bongers RS, Molenaar D, van Hemert S, et al (2009) Lifestyle of *Lactobacillus plantarum* in the mouse caecum. Environ Microbiol 11: 2747–2757. 19638173 10.1111/j.1462-2920.2009.02001.x PMC2978903

[42] Saulnier DM, Molenaar D, de Vos WM, Gibson GR, Kolida S (2007) Identification of prebiotic fructooligosaccharide metabolism in *Lactobacillus plantarum* WCFS1 through microarrays. Appl Environ Microbiol 73: 1753–1765. 17261521 10.1128/AEM.01151-06 PMC1828832

[43] Bolstad BM, Irizarry RA, Astrand M, Speed TP (2003) A comparison of normalization methods for high density oligonucleotide array data based on variance and bias. Bioinformatics 19: 185–193. 12538238 10.1093/bioinformatics/19.2.185

[44] Kampa D, Cheng J, Kapranov P, Yamanaka M, Brubaker S, et al (2004) Novel RNAs identified from an in-depth analysis of the transcriptome of human chromosomes 21 and 22. Genome Res 14: 331–342. 14993201 10.1101/gr.2094104 PMC353210

[45] Crooks GE, Hon G, Chandonia JM, Brenner SE (2004) WebLogo: a sequence logo generator. Genome Res 14: 1188–1190. 15173120 10.1101/gr.849004 PMC419797

