# Supplementary material for: Genome-Wide Prediction and Validation of Sigma70 Promoters in Lactobacillus plantarum WCFS1
Source: PLoS One. 2012 Sep 20;7(9):e45097. doi: 10.1371/journal.pone.0045097 (PMC3447810; doi:10.1371/journal.pone.0045097)
Supplement: Results S1 — Conserved motifs detected by MEME in the upstream regions of protein-encoding genes in L. plantarum WCFS1. (DOCX) [file pone.0045097.s001.docx]

## Results S1 - Conserved motifs detected by MEME in the upstream regions of protein-encoding genes in *L. plantarum* WCFS1.

By comparing the eight motifs (see Figure S1) detected with previously described intergenic sequence elements from other species, we identified one motif as being the consensus Shine-Dalgarno(SD) sequence of bacteria [[1](#_ENREF_25)] and two motifs that resembled typical promoter sequences described for general sigma factor (σ70 or SigA) dependent promoters [[2](#_ENREF_6)] (Figure S1). Notably, the -10 box (detected as part of motif 2) appeared to encompass the so-called extended -10 box that contains an additional conserved guanine located 2 nt upstream of the consensus -10 box and was previously identified in *B. subtilis* [[3](#_ENREF_27)] and *E. coli* [[4](#_ENREF_46)]. Earlier studies in *E. coli* indicated that for optimal promoter activity the presence of an additional conserved guanine (in the extended -10 box) is required for σ^70^-promoters that are composed of less conserved -35 and -10 sequence elements [[4](#_ENREF_46)].

Another motif resembled the binding site for CcpA, known as the catabolite repression element (CRE) [[5](#_ENREF_47)]. CcpA functions as a general regulatory protein for catabolite repression in most Gram-positive organisms (for a review see [[6](#_ENREF_48)]) and was predicted to regulate approximately 200 proteins in *L. plantarum* (based on a motif search performed with the motif described by [[7](#_ENREF_49)]). Using the motif identified in this study we found 276 occurrences of CRE (p-value < 10^-5^). In addition, two motifs were related to transposon elements, as they only appeared in the upstream regions of so-called transposases, while one motif resembled typical T-box regulatory elements that were reported before for *L. plantarum* WCFS1 [[8](#_ENREF_50)]. Finally, our analyses also uncovers a novel motif for which no apparent role could be assigned (Figure S1), but which did not resemble the previously reported *L .plantarum*-specific LPSMs [[9](#_ENREF_51)].

# References

1. Shine J, Dalgarno L (1975) Determinant of cistron specificity in bacterial ribosomes. Nature 254: 34-38.

2. deHaseth PL, Zupancic ML, Record MT Jr (1998) RNA polymerase-promoter interactions: the comings and goings of RNA polymerase. J Bacteriol 180: 3019-3025.

3. Helmann JD (1995) Compilation and analysis of *Bacillus subtilis* sigma A-dependent promoter sequences: evidence for extended contact between RNA polymerase and upstream promoter DNA. Nucleic Acids Res 23: 2351-2360.

4. Mitchell JE, Zheng D, Busby SJ, Minchin SD (2003) Identification and analysis of 'extended -10' promoters in *Escherichia coli*. Nucleic Acids Res 31: 4689-4695.

5. Miwa Y, Nakata A, Ogiwara A, Yamamoto M, Fujita Y (2000) Evaluation and characterization of catabolite-responsive elements (cre) of *Bacillus subtilis*. Nucleic Acids Res 28: 1206-1210.

6. Warner JB, Lolkema JS (2003) CcpA-dependent carbon catabolite repression in bacteria. Microbiol Mol Biol Rev 67: 475-490.

7. Siezen R, Boekhorst J, Muscariello L, Molenaar D, Renckens B, et al. (2006) *Lactobacillus plantarum* gene clusters encoding putative cell-surface protein complexes for carbohydrate utilization are conserved in specific gram-positive bacteria. BMC Genomics 7: 126.

8. Wels M, Groot Kormelink T, Kleerebezem M, Siezen RJ, Francke C (2008) An in silico analysis of T-box regulated genes and T-box evolution in prokaryotes, with emphasis on prediction of substrate specificity of transporters. BMC Genomics 9: 330.

9. Wels M, Bongers RS, Boekhorst J, Molenaar D, Sturme M, et al. (2009) Large intergenic cruciform-like supermotifs in the *Lactobacillus plantarum* genome. J Bacteriol 191: 3420-3423.
