# Supplementary material for: Genome-Wide Prediction and Validation of Sigma70 Promoters in Lactobacillus plantarum WCFS1
Source: PLoS One. 2012 Sep 20;7(9):e45097. doi: 10.1371/journal.pone.0045097 (PMC3447810; doi:10.1371/journal.pone.0045097)
Supplement: Results S2 — Interpretation of TAR detection results. (DOCX) [file pone.0045097.s002.docx]

## Results S2 - Interpretation of TAR detection results.

From tiling-array derived transcription data, 1167 transcriptionally active regions (TARs) were detected using a global threshold approach (see Methods). The 5’-ends of TARs may be the result of transcription initiation but can also arise by other events, such as degradation, processing or cross-hybridisation. Since 5’-ends of TARs were used to infer the position of TSSs, we carefully studied the TAR detection results and observed that the resolution of the tiled micro array and the measured signal intensity compared to the background signal of the tiling array data influence our TSS prediction results. We have selected two examples to illustrate these limitations of the chosen approach.

Of the 1167 TARs, 912 contain genes. Of these TARs, 434 were preceded by a single putative TSS and encompassed several genes (two or more genes), probably representing operons (Figure S2). For example, *lp_1273* and *lp_1274* appeared to be transcribed in a single TAR and at a similar level, supporting their genetic organisation in an operon (Figure S2). Notably, for this particular operon, the algorithm assigned its TSS 537 nt upstream of the 5’-end of *lp_1273*. More detailed inspection of the signal intensity pattern of the *lp_1273-1274* operon illustrates a potential pitfall encountered in promoter validation using tiling microarrays (Figure S2). While the assigned TSS upstream of *lp_1273* is clearly correlated with a predicted σ^70^-promoter located 27 nt upstream of this TSS, a second predicted σ^70^-promoter is located further downstream within this TAR at position -97 nt relative to the start codon of *lp_1273*. Moreover, the probe signals mapped to the genomic region of this second predicted promoter were significantly lower compared to those of surrounding probes, leading to an apparent drop in transcription intensity. This detailed analysis may indicate that this TAR is actually composed of two adjacent TARs that are both expressed at a significant level and that the quantitative dynamic range of the tiling array expression data is insufficient to separate them. Alternatively, the region is transcribed as a single, 5’-extended transcript that encompasses regions of lower stability (e.g., through endonuclease-mediated degradation) and/or hybridization efficiency.

Another example is illustrated in Figure S3. The measured signal intensity suggests that *lp_1541* and *lp_1543* are both transcribed at different expression levels. The expression level of *lp_1543* is low compared to *lp_1541*. While a TSS upstream of *lp_1541* is clearly correlated with a predicted σ^70^-promoter located 2 nt upstream of this TSS, the putative TSS of *lp_1543* is located 145 nt downstream of the annotated translation start of *lp_1543* and a σ^70^-promoter is predicted 31 nt upstream of this translation start. Inferring the TSS position based on the 5’-end of the TAR failed in this case because *lp_1543* is low expressed which makes it difficult to infer a clear TSS. It is also possible that some other (regulatory) effects cause a short transcript leading to an alternative TSS.

In the cases mentioned above the tiling array design and methodology employed here is not suitable to confidently distinguish between these possible explanations which may lead to the false-positive identification of TSS.
