# Supplementary material for: Genome-Wide Prediction and Validation of Sigma70 Promoters in Lactobacillus plantarum WCFS1
Source: PLoS One. 2012 Sep 20;7(9):e45097. doi: 10.1371/journal.pone.0045097 (PMC3447810; doi:10.1371/journal.pone.0045097)
Supplement: Results S3 — Evaluation of results of the TSS prediction method. (DOCX) [file pone.0045097.s003.docx]

## Results S3 - Evaluation of results of the TSS prediction method.

The performance of the TSS prediction method was evaluated using experimentally determined TSSs. Recent publications were selected that report experimentally determined transcription start sites (TSSs) in *Lactobacillus plantarum*. In all publications, TSSs were determined by primer extensions on total RNA from *L. planatrum* grown on rich medium (MRS or CDM). If the TSSs were determined in strains of this species other than *L. planatrum* WCFS1, sequence information on the TSSs were used to locate their genomic positions in genome sequence of *L. planatrum* WCFS1 reference. In the table below, the genomic positions of the TSSs of 15 genes determined by primer extension are provided. They were compared to the TSSs determined in this study. For three genes, TSSs could not be assigned on basis of the tiling array data because the expression signals were too low to detect any transcript, which is most likely due to the culture conditions employed. For the other 12 genes, the differences of genomic positions of the TSSs determined by primer extension and by tiling array data for 11 genes were smaller than 28 nt, likely within the resolution of our tiling probe design (probes were 14 nt apart) whereas one gene showed a difference of more than 50 nt. Out of these 12 genes, 8 genes were highly expressed in our experimental data (averaged expression signal belonged to the upper 40% percentile of the averaged signals of all TARs). The references to the publications in which the primer extension results were reported are listed below.

**Comparison of TSS positions determined by primer extension and inferred from tiling-array data.**

| **Gene** | **TSS position (in nt), primer extension** | **TSS position (in nt), tiling array** | **Absolute difference (in nt)** | **Expression** | **Reference** |
| --- | --- | --- | --- | --- | --- |
| groES (lp_0727) | 664949 | 664930 | 19 | Highly expressed | 1 |
| cggR (lp_0788) | 723751 | 723765 | 14 | Highly expressed | 2 |
| ftsH (lp_0547) | 496342 | 496337 | 5 | Highly expressed | 3 |
| mntH2 (lp_2992) | 2661975 | 2661999 | 24 |  | 4 |
| padA (lp_3665) | 3286749 | 3286766 | 17 | Highly expressed | 5 |
| padR (lp_3664) | 3286727 | 3286741 | 14 | Highly expressed | 5 |
| bglH (lp_3525) | 3141965 | 3141944 | 21 |  | 6 |
| flmA (lp_0593) | 567863 | - |  | No expression | 7 |
| flmB (lp_0290) | 263020 | - |  | No expression | 7 |
| flmC (lp_1000) | 924045 | 923991 | 54 |  | 7 |
| malE (lp_0175) | 151184 | - |  | No expression | 8 |
| ccpA (lp_2256) | 2040383 | 2040394 | 11 | Highly expressed | 9 |
| pyrR1 (lp_2696) | 2407985 | 2408011 | 26 |  | 10 |
| Hsp1 (lp_0129) | 114959 | 114986 | 27 | Highly expressed | 11 |
| Hsp3 (lp_3352) | 2982027 | 2982050 | 23 | Highly expressed | 11 |

**References**

1. Castaldo C, Siciliano RA, Muscariello L, Marasco R, Sacco M (2006) CcpA affects expression of the groESL and dnaK operons in *Lactobacillus plantarum*. Microb Cell Fact 5: 35.

2. Castaldo C, Vastano V, Siciliano RA, Candela M, Vici M, et al. (2009) Surface displaced alfa-enolase of *Lactobacillus plantarum* is a fibronectin binding protein. Microb Cell Fact 8: 14.

3. Fiocco D, Collins M, Muscariello L, Hols P, Kleerebezem M, et al. (2009) The *Lactobacillus plantarum* ftsH gene is a novel member of the CtsR stress response regulon. J Bacteriol 191: 1688-1694.

4. Groot MN, Klaassens E, de Vos WM, Delcour J, Hols P, et al. (2005) Genome-based in silico detection of putative manganese transport systems in *Lactobacillus plantarum* and their genetic analysis. Microbiology 151: 1229-1238.

5. Gury J, Barthelmebs L, Tran NP, Divies C, Cavin JF (2004) Cloning, deletion, and characterization of PadR, the transcriptional repressor of the phenolic acid decarboxylase-encoding padA gene of *Lactobacillus plantarum*. Appl Environ Microbiol 70: 2146-2153.

6. Marasco R, Muscariello L, Varcamonti M, De Felice M, Sacco M (1998) Expression of the bglH gene of *Lactobacillus plantarum* is controlled by carbon catabolite repression. J Bacteriol 180: 3400-3404.

7. Muscariello L, Marasco R, De Felice M, Sacco M (2001) The functional ccpA gene is required for carbon catabolite repression in *Lactobacillus plantarum*. Appl Environ Microbiol 67: 2903-2907.

8. Muscariello L, Vastano V, Siciliano RA, Sacco M, Marasco R (2011) Expression of the *Lactobacillus plantarum* malE gene is regulated by CcpA and a MalR-like protein. J Microbiol 49: 950-955.

9. Muscariello L, Marino C, Capri U, Vastano V, Marasco R, et al. (2012) CcpA and three newly identified proteins are involved in biofilm development in *Lactobacillus plantarum*. J Basic Microbiol.

10. Nicoloff H, Elagoz A, Arsene-Ploetze F, Kammerer B, Martinussen J, et al. (2005) Repression of the pyr operon in *Lactobacillus plantarum* prevents its ability to grow at low carbon dioxide levels. J Bacteriol 187: 2093-2104.

11. Spano G, Capozzi V, Vernile A, Massa S (2004) Cloning, molecular characterization and expression analysis of two small heat shock genes isolated from wine *Lactobacillus plantarum*. J Appl Microbiol 97: 774-782.
