# Supplementary figures and images for: Genome-Wide Prediction and Validation of Sigma70 Promoters in Lactobacillus plantarum WCFS1
Source: PLoS One. 2012 Sep 20;7(9):e45097. doi: 10.1371/journal.pone.0045097 (PMC3447810; doi:10.1371/journal.pone.0045097)

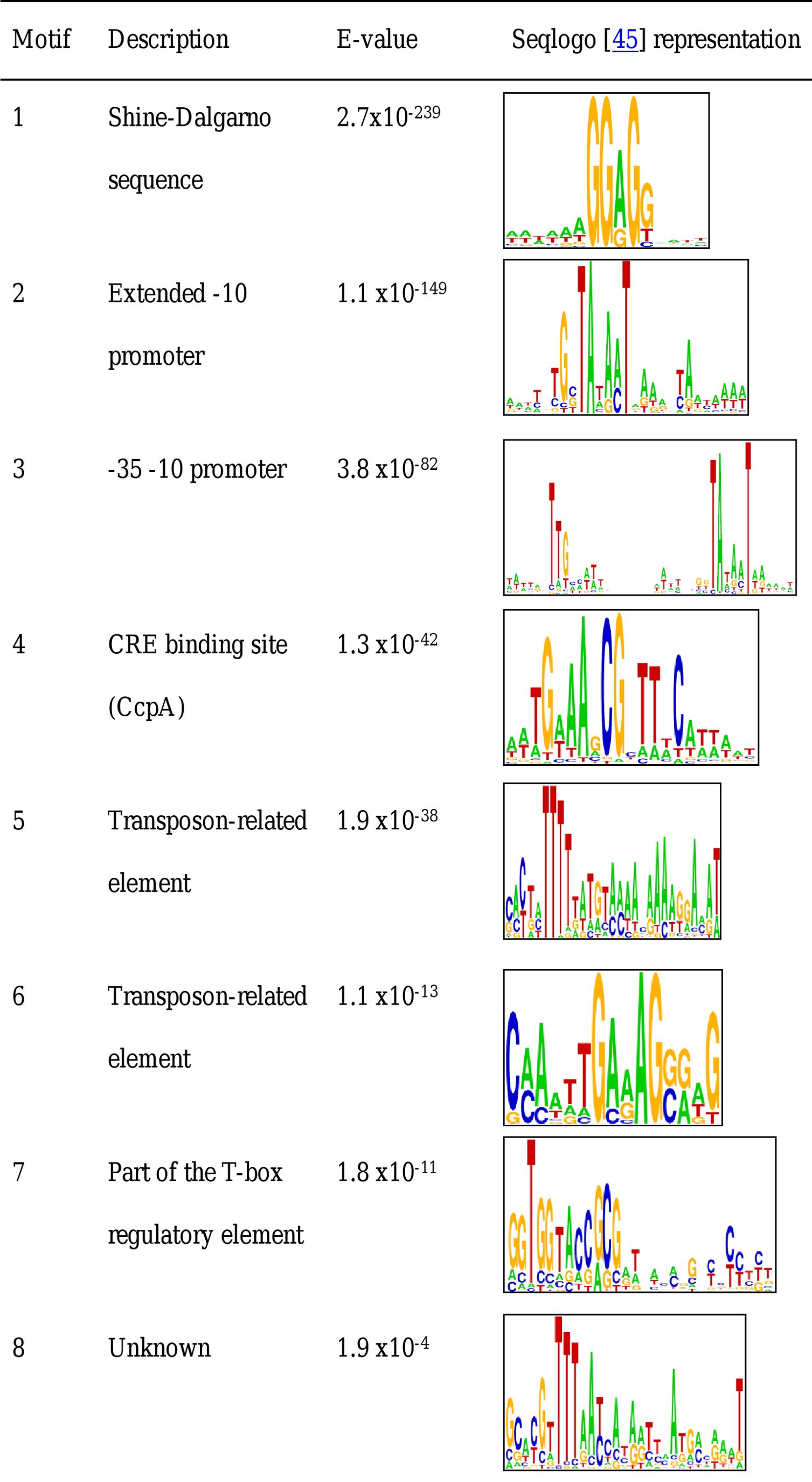

Supplement: Figure S1 — Motifs detected by MEME in L. plantarum WCFS1. Motifs are displayed as Seqlogo's that contain at each position stacks of letters. The height of an individual letter in a stack represents the probability of the letter at that position in an occurrence of the motif. The position-specific probabilities are retrieved from the position-specific weight matrices calculated by MEME. (TIF) [file pone.0045097.s004.tif]

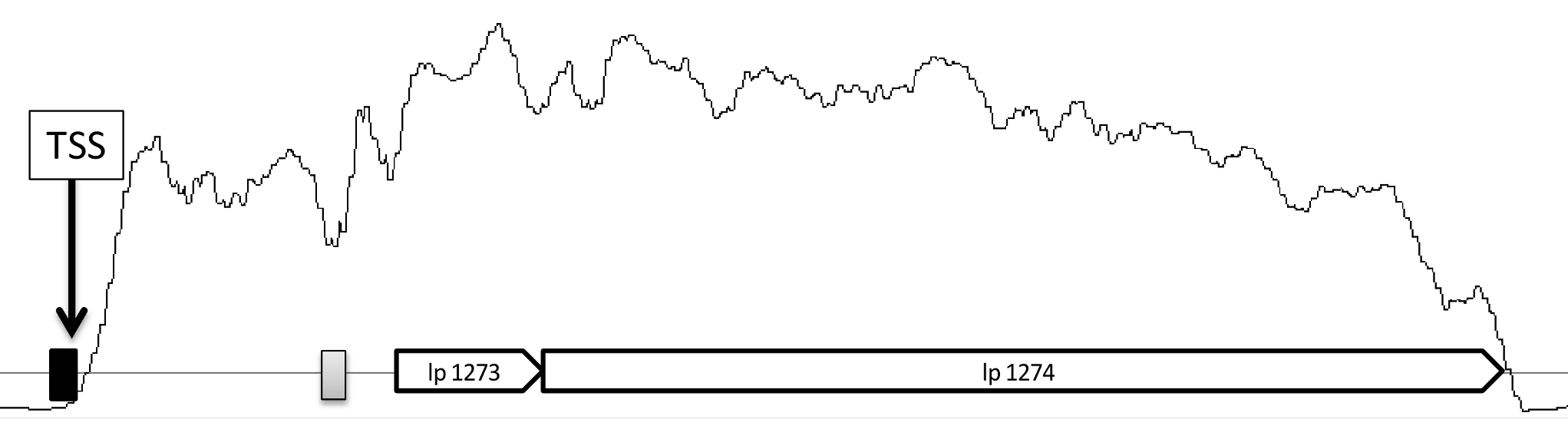

Supplement: Figure S2 — Inferred signal intensity of a genomic region containing two genes. Inferred signal intensity plotted on part of the L. plantarum WCFS1 chromosome (genome location: 1156200–1159500 of the forward strand). At this location two genes are located (lp_1273 and lp_1274) which are considered to form an operon. The tiling microarray signal intensities measured confirms that both genes are transcribed at an approximately equal level and suggest that the inferred transcription start is 537 nucleotides upstream of lp_1273. The black box indicates the position of a predicted σ70-promoter. The grey box indicates the position of a second predicted σ70-promoter with no close TSS. (TIF) [file pone.0045097.s005.tif]

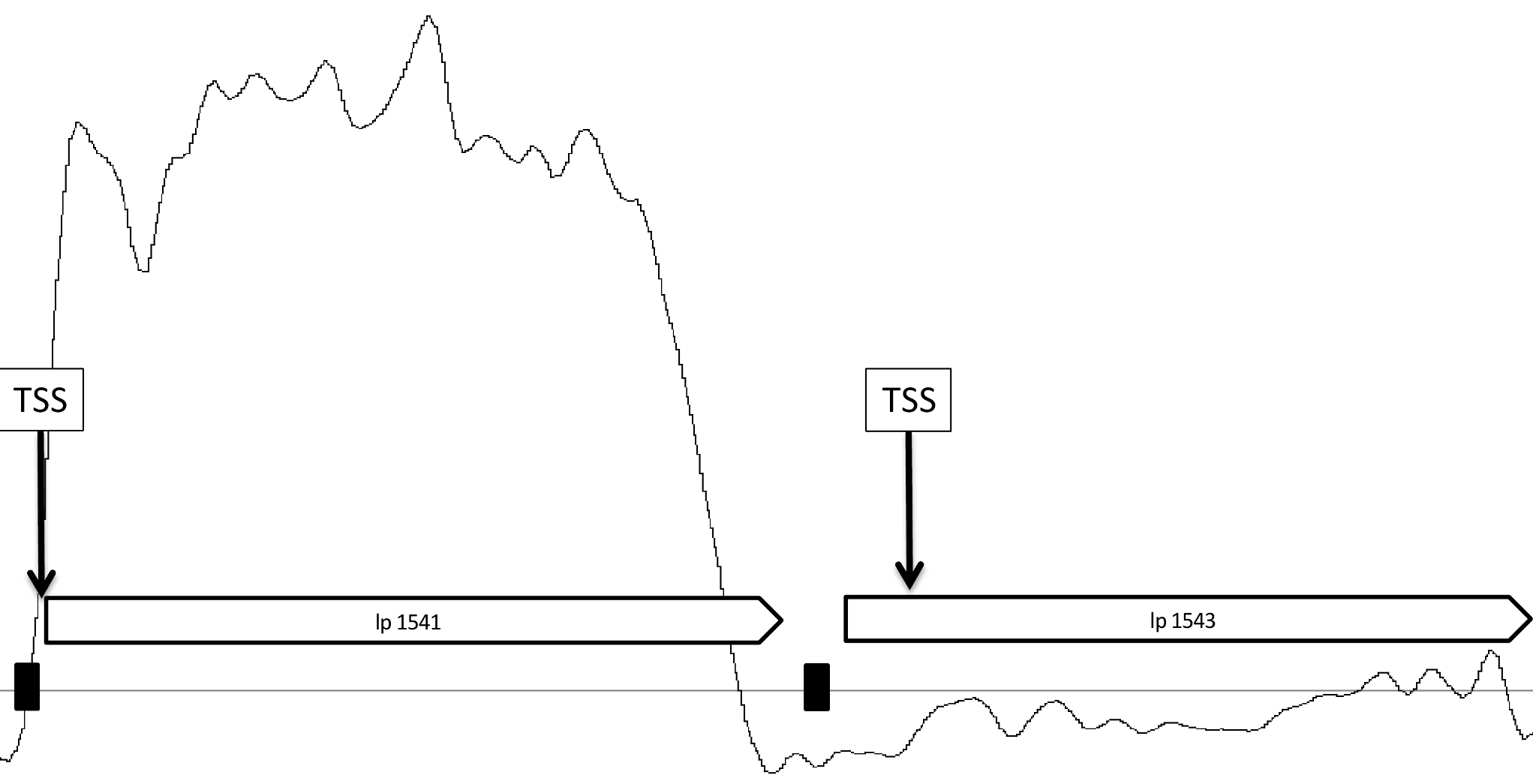

Supplement: Figure S3 — Inferred signal intensity of a genomic region containing two genes. Inferred signal intensity plotted on part of the L. plantarum WCFS1 chromosome (genome location: 1407149–1410217 of the forward strand). At this location two genes are located (lp_1541 and lp_1543) expressed at different levels. For lp_1541 a putative TSS and a predicted σ70-promoter co-localized (2 nt apart) upstream to the annotated translation start of the gene. For lp_1543 a putative TSS is located 145 nt downstream of the annotated translation start of lp_1543 and a σ70-promoter is predicted 31 nt upstream of this translation start. The low signal expression of lp_1543 makes it difficult to infer a clear TSS position. The black boxes indicate the positions of predicted σ70-promoters. (TIF) [file pone.0045097.s006.tif]

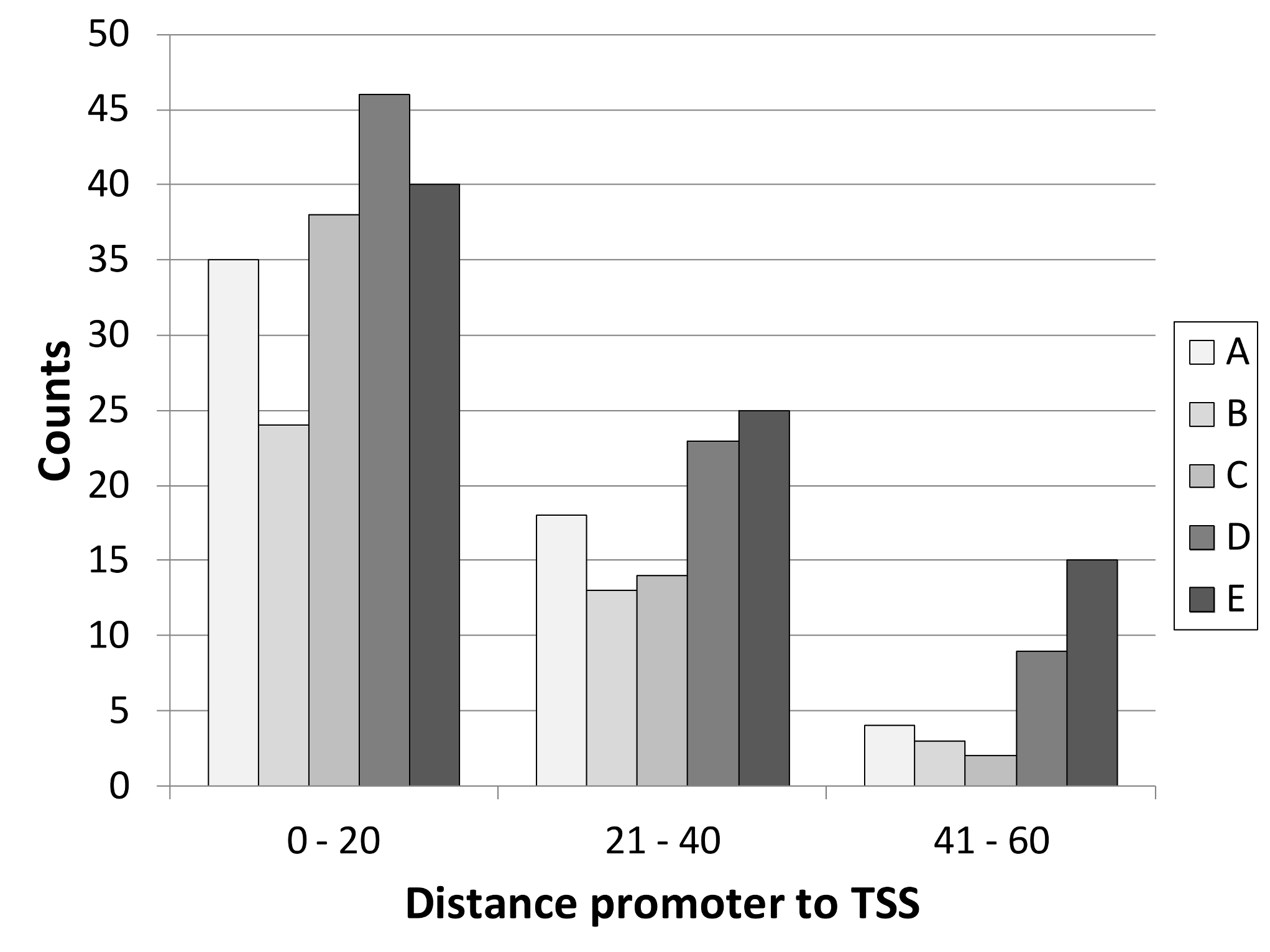

Supplement: Figure S4 — Distribution of distances between promoters and TSSs. Distribution of distances (in nt) for 568 cases where a predicted σ70-promoter (10−4≤p-values<10−3) and a TSS were found in proximity (≤60 nt) of each other. TSSs are divided into 5 groups (A, B, C, D, E) defined by the differences between the means of signal intensities upstream and downstream of the TSS. The 5 groups are the inter-percentile ranges (E: 0–20%, D: 21–40%, C:41–60%, B: 61–80%, A: 81–100%) of the ranked mean differences (see Methods). At distances above 40 nt the observed number of co-localized TSSs and promoters (≤10 counts) is equal to the expected number co-localized TSSs and promoters (see Methods). (TIF) [file pone.0045097.s007.tif]

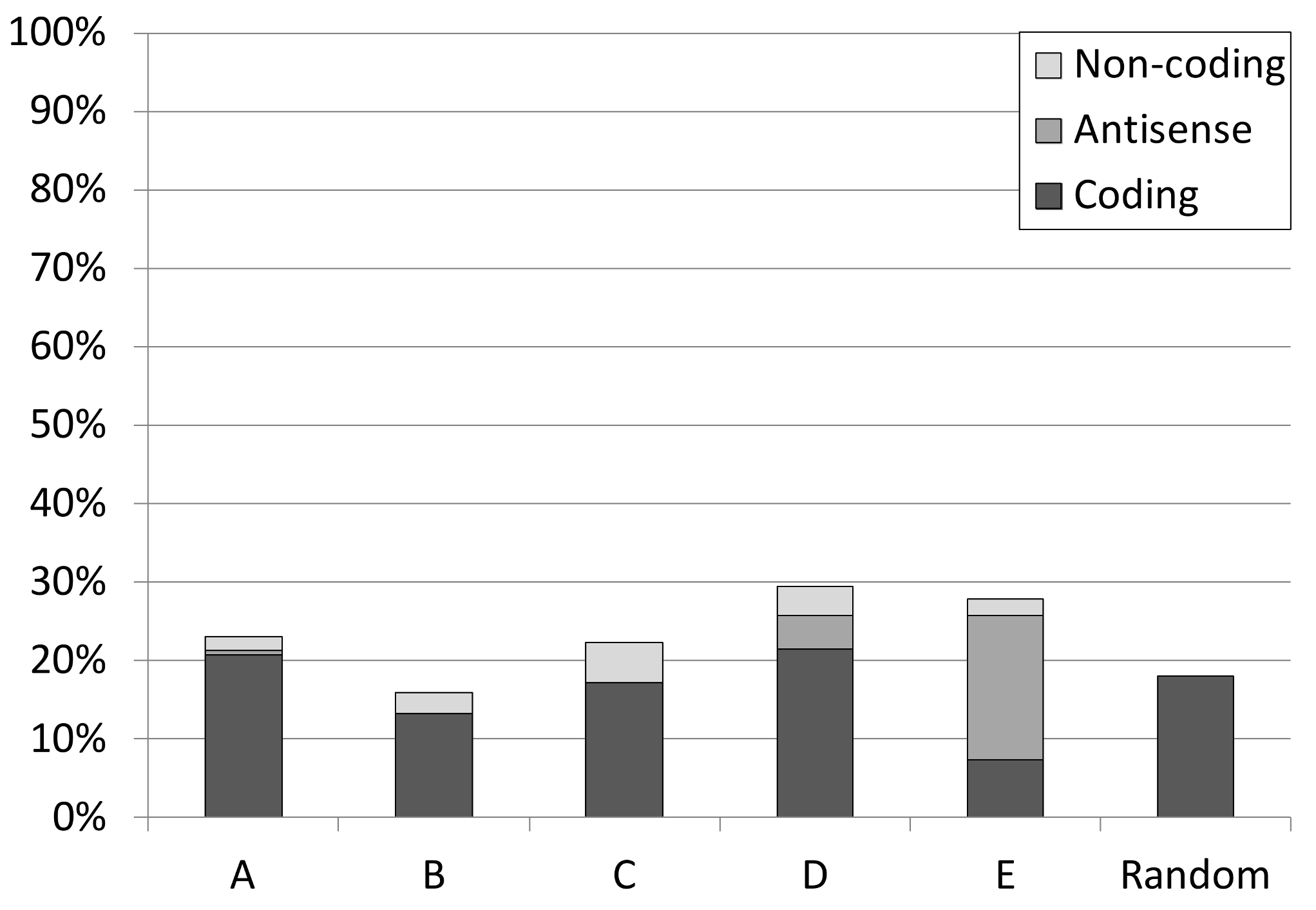

Supplement: Figure S5 — Frequency distribution of validated promoters. Frequency distribution of σ70-promoters (10−4≤p-values<10−3) with TSSs found in proximity (≤40 nt) of each other. Frequencies are given for 5 groups (A, B, C, D, E) and the expected frequency of co-localized TSSs and promoters (Random). The 5 groups are the inter-percentile ranges (E: 0–20%, D: 21–40%, C:41–60%, B: 61–80%, A: 81–100%) of the ranked mean differences between the means of signal intensities upstream and downstream of the TSS (see Methods). (TIF) [file pone.0045097.s008.tif]
